# Countries with Higher Levels of Gender Equality Show Larger National Sex Differences in Mathematics Anxiety and Relatively Lower Parental Mathematics Valuation for Girls

**DOI:** 10.1371/journal.pone.0153857

**Published:** 2016-04-21

**Authors:** Gijsbert Stoet, Drew H. Bailey, Alex M. Moore, David C. Geary

**Affiliations:** 1 School of Education, University of Glasgow, Glasgow, Scotland, United Kingdom; 2 School of Education, University of California Irvine, Irvine, CA, United States of America; 3 Department of Psychological Sciences, University of Missouri, Columbia, MO, United States of America; Goethe-Universitat Frankfurt am Main, GERMANY

## Abstract

Despite international advancements in gender equality across a variety of societal domains, the underrepresentation of girls and women in Science, Technology, Engineering, and Mathematics (STEM) related fields persists. In this study, we explored the possibility that the sex difference in mathematics anxiety contributes to this disparity. More specifically, we tested a number of predictions from the prominent gender stratification model, which is the leading psychological theory of cross-national patterns of sex differences in mathematics anxiety and performance. To this end, we analyzed data from 761,655 15-year old students across 68 nations who participated in the Programme for International Student Assessment (PISA). Most importantly and contra predictions, we showed that economically developed and more gender equal countries have a lower overall level of mathematics anxiety, and yet a larger national sex difference in mathematics anxiety relative to less developed countries. Further, although relatively more mothers work in STEM fields in more developed countries, these parents valued, on average, mathematical competence more in their sons than their daughters. The proportion of mothers working in STEM was unrelated to sex differences in mathematics anxiety or performance. We propose that the gender stratification model fails to account for these national patterns and that an alternative model is needed. In the discussion, we suggest how an interaction between socio-cultural values and sex-specific psychological traits can better explain these patterns. We also discuss implications for policies aiming to increase girls’ STEM participation.

## Introduction

Historically, girls have had fewer educational opportunities than boys, especially within the domains of Science, Technology, Engineering, and Mathematics (STEM)[1]. Through changes in social attitudes, especially in highly developed nations, opportunities have improved and girls’ and women’s participation in STEM subjects has increased, although not to the level of boys’ and men’s participation. The exact reasons for this disparity in participation are currently unknown; while some researchers have vigorously argued that girls are still negatively affected by gender-specific stereotypes [2–5], others have argued that most structural barriers keeping girls out of STEM have now been removed [6]. Apart from these social factors, however, a variety of psychological factors may contribute to the avoidance of these academic domains in general, as well as contribute to the continued underrepresentation of women in these fields (e.g., [7, 8]). In particular, we focus on the potential contributions of sex differences in mathematics anxiety to the lack of equal representation in STEM pursuits. (We use the word “sex” to refer to the sex of participants, male or female. In this study, we do not distinguish between the concepts “sex” and “gender” as some social scientists do, and both these terms could be used interchangeably in the context of our paper.)

In terms of performance, girls score lower than boys on mathematics tests in most developed nations [9]. While the overall international average between boys and girls is relatively small (around 0.12 standard deviation), the difference is larger among higher achieving students [9–11]. And indeed, there are few women among the top performers in mathematics [9, 12–14]. While some researchers have repeatedly stated that the sex difference in mathematics performance is negligibly small [15, 16] (the view that sex differences are mostly very small or non-existent is most prominently expressed in “the gender similarity hypothesis” [17], for a critical response see [18]), it is nonetheless the case that this difference is relevant; one of the main psychological and educational research aims is to determine which factors can explain the sex difference in mathematics performance (which is reflected in the large number of studies on this topic published each year). Thus even when overall sex differences in mean levels of mathematics performance are relatively small, there is a continuing debate about these differences. Moreover, the magnitude of these differences increases with increases in levels of performance, which is more relevant to STEM participation than are differences at the mean.

Mathematics anxiety is a psychological factor that can undermine the pursuit of mathematics, and refers to the negative feelings (affect) experienced during the preparation of and during explicit engagement in mathematical pursuits. This construct is related to a host of negative academic outcomes, including lower enjoyment in the domain, lower intent to pursue and excel in mathematics, lower mathematics-related self-efficacy, and poorer mathematical achievement throughout the academic career [19–25]. As such, individuals who report experiencing mathematics anxiety are more likely to disengage from practice with mathematical concepts and procedures, which could have negative long-term economic consequences for them, including fewer career prospects and lower earning potential relative to those who do not experience mathematics anxiety [26–28].

Mathematics anxiety has a neural signature that distinguishes it from other non-cognitive constructs (e.g., self-concept) that could influence engagement in mathematics. (Non-cognitive variables often have a cognitive component, and the distinction between cognitive and non-cognitive variables is not an all-or-none distinction [29]). Recent neuroimaging studies reveal that increases in self-reported mathematics anxiety are associated with neural activation patterns suggestive of learned fear responses in young children [25, 30], and also that the simple anticipation of mathematical problem solving (in contrast to anticipation of language-based problem solving) is neurally equivalent to the anticipation of physical harm in adults [31]. Thus, while related to various other psychological constructs it appears that mathematics anxiety is a conceptually and empirically distinct phenomenon that represents a true negative emotional, even fearful, response to mathematical pursuits [32, 33]; this reaction to mathematics is believed to foster active avoidance of the domain, and by extension avoidance of STEM fields that are highly reliant on mathematical skills [28].

Importantly, it is well established that girls and women report greater trait mathematics anxiety than do boys and men [2, 21, 34–39], which may contribute to the lower participation of women than men in college majors and career paths that involve mathematics (e.g., [7]). Given recent interest in the examination of teacher and parental influences on the development of mathematics anxiety [7, 40], our study focuses specifically on the question of how sex differences in mathematics anxiety are related to societal and family variables. We do so using data from the Programme for International Student Assessment, PISA, the world’s largest international comparison of student achievement in 15-year olds [41, 42] (see Materials and Methods for details).

### Sex differences in mathematics anxiety

The study of mathematics anxiety has both theoretical and practical significance. Theoretically, mathematics anxiety lies at the intersection of cognition and affect; it is anxiety about one’s cognitive aptitude and performance within the mathematics domain and can be distinguished from generalized anxiety [25]. Practically, reducing mathematics anxiety has the potential to increase engagement with mathematics and so might indirectly increase the diversity of the STEM workforce (e.g., [43–45]). The general idea is that girls do not perform as well as they could and participate less in STEM, in part, because of their higher levels of mathematics anxiety (compared to boys).

Various surveys and academic studies have reported that the average level of mathematics anxiety is higher in some countries than others [15, 41, 42]. This cross-national variation may provide useful insights into the factors underlying the development of mathematics anxiety. Indeed, many researchers have argued that certain social and cultural factors might exacerbate girls’ and women’s mathematics anxiety and undermine their mathematical performance (e.g., [7]).

A prominent version of the argument that social and cultural factors negatively affect women’s mathematics performance and affect is the gender stratification hypothesis (or theory) [15, 46–48]. The prominence of this hypothesis is reflected, for example, in the fact that the papers by Else-Quest et al. [15] and by Guiso et al. [48] are marked as highly cited papers in the Web of Science database and has generally influenced academic’s opinion about gender equality (e.g., [49]). The essence of the hypothesis is that the observed sex differences in performance and affect are the result of a lack of societal opportunities (e.g., in education, access to resources, finances, etc). The core prediction is that sex differences in psychological abilities, affect, and outcomes will fade as social barriers to women’s participation disappear—that is, as social beliefs regarding historically male-dominated domains fade and opportunities for men and women become more equal. One key mechanism is children’s relationship with their parents, including expectations and beliefs about the mathematical potential of boys and girls, and the number of mothers serving as role models for their daughters within the STEM fields [15]. Else-Quest and colleagues [15] tested associated predictions of this hypothesis using the 2003 PISA, and found that *the higher* the proportion of women employed in a country’s research sector *the smaller* the sex differences in mathematics achievement and mathematics anxiety.

At the same time, the gender-stratification hypothesis fails to account for important empirical findings. For example, Else-Quest and colleagues [15] reported that girls in the 2003 PISA data had relatively *higher* levels of mathematics anxiety than boys in more gender equal countries, contra the hypothesis. In other words, girls in highly gender equal countries, such as Norway and Germany, have relatively higher levels of mathematics anxiety than do boys in those countries, whereas girls and boys in less gender equal countries, such as Mexico and Italy, do not differ as much in mathematics anxiety. Else-Quest et al. [15] dealt with this contradiction by proposing the addition of a number of auxiliary hypotheses to the gender-stratification model (details below).

### How the gender stratification model can be tested

Else-Quest and colleagues [15] offered two possibilities to explain their finding that sex differences in mathematics anxiety are larger in more gender equal countries, both of which we explicitly test (note that at the time of their study, the much larger and more detailed 2012 PISA data set analyzed here was not yet available, and these predictions could not have been tested). The first relates to the idea that more gender-equal countries tend to have lower levels of power distance (Hofstede, 1980), which captures the extent of between-strata social comparisons. As such, Else-Quest and colleagues [15] reasoned that higher gender-equality and smaller power distance would lead girls to compare themselves to boys more than in situations with less gender-equality and larger power distance. The heightened between-sex comparisons would then increase girls’ mathematics anxiety in more gender-equal countries and result in larger sex differences. This is an intriguing idea, but one that was not explicitly tested by Else-Quest and colleagues [15]. We do so here by examining the relation between national indicators of gender equality and sex differences in mathematics performance and anxiety and by contrasting boys and girls from single-sex (less between-sex comparison) and mixed-sex (more between-sex comparison) schools. We reasoned that schools are the main contexts within which cross-sex comparisons would occur for mathematics and thus students in same-sex schools should have fewer opportunities to make those comparisons than students in mixed-sex schools.

The second explanation for why the sex differences in mathematics anxiety are larger in more gender equal countries is that mathematics anxiety grows in prevalence when other more basic needs are satisfied, “That is, the experience of math attitudes and affect may be a luxury, most often experienced by individuals who are not preoccupied with meeting more basic needs” [15]. In the current study, we test this explanation using the same proxy used by Else-Quest and colleagues [15] for development (gender equality) and with an additional more direct measure of basic-needs satisfaction (see below).

Another key issue raised by the gender-stratification hypothesis is the mechanism through which children are socialized to be attracted or averse to mathematics. Some have argued for the importance of girls modeling the behaviors, attitudes, and affect they observe within their families: “if girls’ mothers, aunts, and sisters do not have STEM careers, they will perceive that STEM is a male domain and thus feel anxious about math, lack the confidence to take challenging math courses, and underachieve on math tests” [15]. One difficulty with this explanation is that many of the countries with the highest percentages of women in research fields score poorly on indicators of gender equality. Moreover, the countries that drive the correlation between the proportion of women in research and mathematics performance are a few less wealthy countries with below median overall mathematics performance (namely, Latvia, Thailand, Tunisia, and Serbia; [50]). Further, while previously used measures of women in research fields surely include many women in STEM careers, not all of the women included in these figures are in STEM careers, or in STEM fields in which men are historically overrepresented. A more direct test would be an assessment of the relation between girls’ mathematics anxiety and family members’ occupations. We do so by testing the prediction that the proportion of mothers to fathers in our sample working in STEM will influence sex differences in mathematics anxiety and performance.

In all, we used the data from the two PISA surveys (see Materials and Methods) that focused on both mathematics performance and attitudes towards mathematics; the combination of which enables a more thorough evaluation of the gender-stratification hypotheses than previously possible. The large, diverse sample of students from throughout the world provides an ideal dataset for addressing the important issues raised here. Further, we use the data from the Global Gender Gap Report (see Methods), which provides a prominent international comparison of country-level gender equality, and from the Human Development Report [51], which provides data on the level to which society satisfies basic human needs. In addition to testing the gender-stratification hypothesis, we thoroughly document the empirical relations among mathematics performance, mathematics anxiety, country-level gender equality, and beliefs (of parents and students) about the relative importance of mathematics for boys and girls.

It should also be stressed that the current study focuses predominantly on country-level comparisons, for two major reasons. First, country comparisons are generally effective in testing hypotheses about the influence of socio-cultural factors on human behavior, attitudes, and affect. This because socio-cultural factors can differ considerably between countries, and if it is hypothesized that specific socio-cultural factors influence behavior, attitudes, and affect, this should be reflected in between-country variation in behavior, attitudes, and affect. The second reason is related to policy making; specifically, policy makers can learn from and potentially adopt successful educational policies from other countries. And indeed, PISA has had a major influence on policy making since the first reports were published in the early 2000s [52, 53].

### Research questions

Our first question is whether it is indeed the case that increased levels of development and associated basic-need satisfaction will be associated with increased levels of mathematics anxiety (across countries), based on the argument of Else-Quest and colleagues [15]; as noted above, “the experience of math attitudes and affect may be a luxury, most often experienced by individuals who are not preoccupied with meeting more basic needs. This pattern of findings points to the importance not only of gender equity but also of human development”. This is an important hypothesis of why sex differences in mathematics anxiety are larger in more gender equal and developed nations, and we test this possibility for the first time here.

Our second question tests the degree to which mathematics anxiety is a function of mathematics performance, and to what degree the relation between the national level of mathematics anxiety and development still holds when this variable is taken into account. The rationale for this question is that mathematics anxiety, to some degree, reflects a student’s own reflection on actual performance. To what degree are relations between sex differences in mathematics anxiety and gender equality actually reflecting differences in mathematics performance?

Our third question relates to the use of the power distance hypothesis (as described above) to explain why sex differences in mathematics anxiety are larger in nations with higher levels of gender equality. It is important to note that power distance is only indirectly relevant. What matters for the gender stratification model is that higher levels of power distance are associated with less social interaction between the two sexes [15]. One potential consequence, as described earlier, is a higher level of mathematics anxiety for children in countries with a lower power distance, simply because they have more between-sex interactions and thus more opportunity to between-sex comparisons in mathematics. We evaluate this idea in a more direct way by testing if children in single-sex schools experience lower levels of mathematics anxiety than those in mixed-sex schools (see above for details of the logic behind this).

Our fourth question relates to the influence of parent/child interactions on children’s mathematics anxiety. We test the prediction that the proportion of mothers to fathers working in STEM is negatively related to sex differences in mathematics performance and mathematics anxiety. If this is the case, it would provide a strong corroboration of aspects of the the gender-stratification hypothesis.

## Materials and Method

### Data sources

The 2003 and 2012 PISA data sets were chosen because of their focus on mathematics achievement, attitudes, and affect. PISA is the largest international evaluation of educational performance of 15-year old students in member and partner countries of the Organization for Economic Co-Operation and Development (OECD). Examples of participating economic regions are Hong Kong, Macau, and Shanghai.

We used data from other sources as measures of national gender equality and development. The Global Gender Gap Index (GGI) is a widely used measure of gender equality, based on a number of relevant measures, including levels of education, health, as well as economic and political participation [54]. This report has been published annually since 2006 (i.e., there is no matching data set for 2003). The advantages of GGI over other measures have been discussed elsewhere, and the GGI has been used in previous analyses for comparisons with the 2003 PISA data (e.g., [15]). For our analyses, we used the GGI of the years 2006 and 2012, although changes in relative standing in GGI over time are small; for comparison, the correlation between the GGI data of 2006 and 2009, a three year difference for the countries participating in 2003 and 2009 PISA, is *r*(35) = .95, *p* < .001, and between 2006 and 2012 for the countries participating in the 2003 and 2012 PISA surveys, it is *r*(34) = .94, *p* < .001.

GGI is not only used as a measure of gender equality, but also used as a proxy for the satisfaction of basic needs [15]. As an additional and more direct measure of the satisfaction these needs, we also used the United Nations Human Development Index (HDI, United Nations Development Programme, 2013). The HDI is a composite measure of a number of indicators of the quality of living conditions, including life expectancy and health, education, and financial means. We have GGI for 37 and HDI for 38 of the 41 participating nations and regions in the 2003 PISA. The HDI and GGI are correlated, *r*(35) = .59, *p* < .001. Similarly for the 2012 PISA dataset, the correlation between GGI (of 57 countries) and HDI (of 61 countries) is similar, *r*(55) = .56, *p* < .001.

### Samples

In the 2003 PISA, 276,165 students participated in 41 different countries and economic regions (Table 1). In 2012, 485,490 students in 68 different countries and regions participated in PISA, including all countries that also participated in 2003 (Table 2). In total, our dataset includes 761,655 students. Participating students were between 15 years and 3 months and 16 years and 2 months old. The PISA consortium chooses the participating schools of each country and region. National project managers choose students from these schools [55]. Between 5,000 and 10,000 students from at least 150 schools of each country/region are typically participating in PISA surveys [56].

**Table 1:** For each country, the following information is listed used in the analysis of the 2003 PISA dataset. n = Sample size; GGI = Global Gender Gap; HDI = Human Development Index; PD = Power Distance; math = National mathematics score; mathD = Sex difference in national mathematics score (boys-girls) in standard deviations; anx = National average of mathematics anxiety; anxD = Sex difference in national mathematics score (girls-boys) in standard deviations; EanxD = Sex difference in performance-adjusted national mathematics score (girls-boys) in standard deviations. For the columns mathD, anxD, and EanxD, numbers in bold indicate statistically significant differences.

Country	n	GGI	HDI	PD	math	mathSD	mathD	anx	anxSD	anxD	EanxD
Austria	4597	0.70	0.94	11	506	93	0.08	−0.27	1.15	**0.36**	**0.28**
Australia	12551	0.72	0.96	36	524	95	0.06	−0.05	0.88	**0.31**	**0.23**
Belgium	8796	0.71	0.94	65	529	110	0.07	0.09	0.94	**0.32**	**0.27**
Brazil	4452	0.65	0.79	69	356	100	**0.16**	0.56	0.78	**0.32**	**0.31**
Canada	27953	0.72	0.95	39	532	87	**0.13**	−0.05	1.07	**0.33**	**0.28**
Switzerland	8420	0.70	0.95	34	527	98	**0.17**	−0.29	1.08	**0.44**	**0.39**
Czech Republic	6320	0.67	0.87	57	516	96	**0.16**	−0.04	0.90	**0.26**	**0.25**
Germany	4660	0.75	0.93	35	503	103	**0.09**	−0.26	1.17	**0.38**	**0.34**
Denmark	4218	0.75	0.94	18	514	91	**0.18**	−0.46	1.05	**0.38**	**0.33**
Spain	10791	0.73	0.93	57	485	88	**0.1**	0.28	0.86	**0.34**	**0.29**
Finland	5796	0.80	0.94	33	544	84	**0.09**	−0.32	0.89	**0.39**	**0.29**
France	4300	0.65	0.94	68	511	92	**0.09**	0.33	0.92	**0.39**	**0.31**
Greece	4627	0.65	0.91	60	445	94	**0.21**	0.16	0.95	**0.26**	**0.3**
Hong Kong	4478		0.92	68	550	100	0.04	0.23	0.89	**0.28**	**0.2**
Hungary	4765	0.67	0.86	46	490	94	**0.08**	−0.00	0.89	**0.2**	**0.17**
Iceland	3350	0.78	0.96		515	90	−**0.17**	−0.20	1.07	**0.27**	0.07
Indonesia	10761	0.65	0.70	78	360	81	0.04	0.34	0.64	**0.13**	**0.12**
Ireland	3880	0.73	0.95	28	503	85	**0.17**	0.07	0.93	**0.28**	**0.29**
Italy	11639	0.65	0.93	50	466	96	**0.19**	0.28	0.85	**0.17**	**0.23**
Japan	4707	0.64	0.94	54	534	101	0.08	0.44	1.01	**0.26**	**0.24**
South Korea	5444	0.62	0.90	60	542	92	**0.25**	0.42	0.83	**0.14**	**0.25**
Latvia	4627	0.71	0.84	44	483	88	0.03	0.12	0.78	**0.26**	**0.18**
Liechtenstein	332				536	99	**0.29**	−0.35	0.96	**0.61**	**0.56**
Luxembourg	3923	0.67	0.95	40	493	92	**0.19**	−0.01	1.14	**0.44**	**0.4**
Macao	1250				527	87	**0.24**	0.23	0.98	**0.46**	**0.44**
Mexico	29983	0.65	0.81	81	385	85	**0.13**	0.47	0.73	**0.13**	**0.17**
The Netherlands	3992	0.72	0.94	38	538	93	0.06	−0.38	0.87	**0.38**	**0.29**
Norway	4064	0.80	0.96	31	495	92	0.07	−0.05	1.08	**0.36**	**0.26**
New Zealand	4511	0.75	0.93	22	523	98	**0.15**	−0.10	0.89	**0.31**	**0.27**
Poland	4383	0.68	0.86	68	490	90	0.06	0.04	0.95	0.03	**0.06**
Portugal	4608	0.69	0.90	63	466	88	**0.14**	0.15	0.84	**0.22**	**0.23**
Serbia	4405			86	437	85	0.01	0.28	0.97	−0.04	0
Russia	5974	0.68	0.80	93	468	92	**0.11**	0.14	0.79	**0.16**	**0.17**
Sweden	4624	0.81	0.95	31	509	95	**0.07**	−0.49	0.98	**0.3**	**0.23**
Slovak Republic	7346	0.68	0.85	104	498	93	**0.2**	0.04	0.85	**0.25**	**0.27**
Thailand	5236	0.68	0.78	64	417	82	−0.05	0.48	0.69	**0.11**	0.04
Tunisia	4721	0.63	0.75		359	82	**0.15**	0.61	0.89	**0.35**	**0.34**
Turkey	4855	0.58	0.75	66	423	105	**0.14**	0.35	1.02	**0.2**	**0.22**
Uruguay	5835	0.65	0.84	61	422	100	**0.12**	0.30	0.89	**0.2**	**0.21**
United Kingdom	9535	0.74	0.94	35	508	92	0.07	−0.09	0.92	**0.38**	**0.28**
United States	5456	0.70	0.94	40	483	95	**0.07**	−0.10	1.09	**0.23**	**0.19**

**Table 2:** Data of the 2012 PISA data analysis. Abbreviations as in Table 1 and as follows. ratio1 = the ratio of fathers to mothers with a STEM occupation; ratio2 = as ratio 1 but only for high status STEM occupations, see text for details; pod = sex difference in perceived parental valualation of mathematics in standard deviations; pid = sex difference in actual parental valualation of mathematics in standard deviations.

Country	n	GGI	HDI	PD	math	mathSD	mathD	anx	anxSD	anxD	EanxD	ratio1	ratio2	po	poD	pi	piD
United Arab Emirates	11500	0.64	0.82		434	90	−0.05	0.19	0.98	−**0.08**	−0.08	0.11	0.10	3.39	**0.08**		
Albania	4743	0.67	0.75		394	92	−0.01	0.14	0.90	−0.04	−0.02	0.07	0.13	3.41	0.07		
Argentina	5908	0.72	0.81	49	388	77	**0.18**	0.54	0.86	**0.15**	**0.22**	0.05	0.15	3.26	−0.02		
Austria	4755	0.74	0.90	11	506	92	**0.24**	−0.23	1.14	**0.31**	**0.34**			3.18	**0.3**		
Australia	14481	0.73	0.94	36	504	96	**0.13**	0.03	0.93	**0.36**	**0.3**	0.15	0.24	3.32	**0.22**		
Belgium	8597	0.77	0.90	65	515	102	**0.11**	0.05	0.96	**0.39**	**0.37**	0.12	0.17	3.03	**0.32**	0.16	**0.18**
Brazil	19204	0.69	0.73	69	389	78	**0.21**	0.52	0.77	**0.24**	**0.3**	0.05	0.14	3.39	0.05		
Bulgaria	5282	0.70	0.78	70	439	94	−0.03	0.26	1.00	0.04	0.03	0.21	0.38	3.14	**0.14**		
Canada	21544	0.74	0.91	39	518	89	**0.11**	0.01	1.07	**0.37**	**0.3**	0.10	0.17	3.40	**0.08**		
Switzerland	11229	0.77	0.91	34	531	94	**0.14**	−0.29	1.03	**0.5**	**0.4**	0.24	0.16	3.23	**0.29**		
Chile	6856	0.67	0.82	63	423	81	**0.31**	0.42	0.77	**0.31**	**0.39**	0.11	0.19	3.39	**0.18**	−0.03	−0.03
Colombia	9073	0.69	0.72	67	376	74	**0.34**	0.35	0.80	**0.19**	**0.34**	0.09	0.17	3.33	0.05		
Costa Rica	4602	0.72	0.77	35	407	68	**0.35**	0.40	0.91	**0.44**	**0.51**	0.06	0.13	3.30	**0.15**		
Czech Republic	5327	0.68	0.87	57	499	95	**0.12**	−0.02	0.94	**0.22**	**0.2**	0.16	0.20	2.99	**0.27**		
Germany	5001	0.76	0.92	35	514	96	**0.14**	−0.29	1.14	**0.36**	**0.32**	0.07	0.13	3.30	**0.22**	0.21	**0.23**
Denmark	7481	0.78	0.90	18	500	82	**0.17**	−0.37	1.04	**0.49**	**0.41**	0.17	0.20	3.31	**0.15**		
Estonia	4779	0.70	0.85	40	521	81	**0.07**	−0.16	1.00	**0.2**	**0.17**	0.12	0.29	3.09	**0.17**		
Spain	25313	0.73	0.88	57	484	88	**0.19**	0.21	0.90	**0.32**	**0.32**	0.09	0.17	3.27	**0.11**		
Finland	8829	0.85	0.89	33	519	85	−0.03	−0.32	0.90	**0.43**	**0.27**	0.13	0.18	3.04	**0.09**		
France	4613	0.70	0.89	68	495	97	**0.09**	0.27	0.92	**0.47**	**0.37**	0.12	0.24	3.17	**0.2**		
Greece	5125	0.67	0.86	60	453	88	**0.09**	0.12	0.97	**0.24**	**0.22**	0.10	0.18	3.16	**0.16**		
Hong Kong	4670		0.91	68	561	96	**0.16**	0.13	0.92	**0.37**	**0.33**	0.08	0.06	2.97	0.06	0.07	**0.08**
Croatia	5008	0.71	0.80	73	471	88	**0.13**	0.13	0.98	**0.14**	**0.16**	0.23	0.50	3.15	**0.21**	0.09	**0.09**
Hungary	4810	0.67	0.83	46	477	94	**0.1**	−0.06	0.95	**0.23**	**0.22**	0.16	0.15	3.08	**0.3**	0.08	**0.08**
Iceland	3508	0.86	0.91		493	92	−**0.07**	−0.33	1.06	**0.27**	**0.17**	0.09	0.17	3.39	0.01		
Indonesia	5622	0.66	0.63	78	375	71	0.06	0.28	0.69	0.06	**0.09**	0.03	0.00	3.24	−0.03		
Israel	5055	0.70	0.90	13	466	105	0.11	−0.06	1.05	**0.27**	**0.28**	0.16	0.31	3.45	**0.16**		
Ireland	5016	0.78	0.92	28	501	85	**0.18**	0.11	0.91	**0.35**	**0.35**	0.08	0.12	3.25	**0.2**		
Italy	31073	0.67	0.88	50	485	93	**0.2**	0.31	0.86	**0.25**	**0.29**	0.11	0.24	3.19	**0.09**	0.10	**0.1**
Japan	6351	0.65	0.91	54	536	94	**0.19**	0.37	1.01	**0.3**	**0.34**	0.11	0.10	2.70	**0.1**		
Jordan	7038	0.61	0.70		386	78	−**0.27**	0.51	0.78	−**0.24**	−**0.3**	0.04	0.08	3.29	0.03		
Kazakhstan	5808	0.72	0.75		432	71	0.01	0.03	0.84	−0.06	−0.03	0.21	0.27	3.28	0.02		
South Korea	5033	0.64	0.91	60	554	99	**0.18**	0.31	0.84	**0.26**	**0.28**	0.07	0.08	3.06	**0.18**	0.14	**0.16**
Latvia	4306	0.76	0.81	44	491	82	−0.05	0.02	0.81	0.09	0.07	0.30	1.09	3.04	**0.2**		
Liechtenstein	293		0.88		535	95	**0.24**	−0.27	1.00	**0.45**	**0.41**	0.21	0.18	3.36	0.2		
Lithuania	4618	0.72	0.82	42	479	89	0	−0.07	1.03	**0.19**	**0.12**	0.11	0.27	3.22	**0.2**		
Luxembourg	5258	0.74	0.88	40	490	95	**0.26**	−0.10	1.13	**0.36**	**0.38**	0.08	0.17	3.15	**0.17**		
Malaysia	5197	0.65	0.77	104	421	81	−**0.1**	0.43	0.78	0.01	−0.05	0.08	0.16	3.38	−**0.13**		
Montenegro	4744		0.79		410	83	0	0.18	0.93	0.03	0.05	0.22	0.29	3.11	**0.15**		
Macao	5335				538	94	0.03	0.19	0.99	**0.39**	**0.27**	0.04	0.07	2.89	0.01	0.10	**0.11**
Mexico	33806	0.67	0.78	81	413	74	**0.19**	0.44	0.81	**0.24**	**0.29**	0.06	0.07	3.41	**0.08**	0.02	0.02
The Netherlands	4460	0.77	0.92	38	523	92	**0.11**	−0.39	0.91	**0.28**	**0.28**	0.11	0.20	2.94	**0.19**		
Norway	4686	0.84	0.96	31	489	90	0.02	0.02	1.05	**0.34**	**0.24**	0.25	0.36	3.35	0.03		
New Zealand	4291	0.78	0.92	22	500	100	**0.15**	0.10	0.89	**0.38**	**0.32**	0.12	0.18	3.34	**0.16**		
Peru	6035	0.67	0.74	64	368	84	**0.22**	0.33	0.72	**0.17**	**0.25**	0.02	0.03	3.46	−0.06		
Poland	4607	0.70	0.82	68	518	90	0.04	−0.03	1.03	**0.11**	**0.11**	0.12	0.30	3.12	0.05		
Portugal	5722	0.71	0.82	63	487	94	**0.12**	0.01	0.82	**0.15**	**0.19**	0.12	0.27	3.42	−0.01	0.07	**0.08**
Qatar	10966	0.63	0.83		376	100	−**0.16**	0.27	1.09	−**0.08**	−**0.1**	0.10	0.11	3.25	0.03		
Romania	5074	0.69	0.79	90	445	81	0.05	0.39	0.83	0.01	0.05	0.20	0.27	3.08	0		
Perm (Russia)	1761				484	89	0.08	0.10	0.77	**0.29**	**0.24**	0.32	0.50	3.09	**0.25**		
Serbia	4684	0.70	0.77	86	449	91	**0.1**	0.19	0.95	−0.02	0.05	0.21	0.34	3.03	**0.24**		
Russia	5231	0.70	0.79	93	482	86	−0.02	0.11	0.84	**0.17**	**0.09**	0.27	0.40	3.07	**0.28**		
Sweden	4736	0.82	0.92	31	478	92	−0.03	−0.34	0.99	**0.34**	**0.21**	0.15	0.37	3.24	**0.09**		
Singapore	5546	0.70	0.90	74	573	105	−0.03	0.16	0.92	**0.14**	**0.07**	0.16	0.20	3.42	**0.12**		
Shanghai	5177				613	101	0.06	0.02	0.94	**0.42**	**0.29**	0.33	0.16	3.09	−0.04		
Slovenia	5911	0.71	0.89	71	501	92	0.04	0.07	0.95	**0.17**	**0.13**	0.17	0.24	3.05	**0.15**		
Slovak Republic	4678	0.68	0.84	104	482	101	**0.09**	0.01	0.96	**0.23**	**0.2**	0.20	0.22	2.97	**0.26**		
Thailand	6606	0.69	0.69	64	427	82	−**0.17**	0.51	0.63	**0.11**	−0.05	0.11	0.04	3.25	−**0.09**		
Tunisia	4407		0.71		388	78	**0.19**	0.64	0.86	**0.1**	**0.19**	0.06	0.09	3.29	0.01		
Turkey	4848	0.60	0.72	66	448	91	0.09	0.28	1.04	−0.02	0.05	0.04	0.10	3.30	−**0.09**		
Chinese Taipei	6046			58	560	116	0.05	0.31	0.94	**0.32**	**0.23**	0.18	0.16	2.89	**0.14**		
Uruguay	5315	0.67	0.79	61	409	89	**0.13**	0.36	0.93	**0.22**	**0.28**	0.12	0.42	3.28	**0.15**		
Connecticut	1697				506	99	**0.15**	−0.24	1.03	**0.32**	**0.29**	0.08	0.16	3.24	0.12		
Florida	1896				467	85	**0.17**	−0.05	1.11	**0.34**	**0.34**	0.09	0.21	3.26	0.06		
United Kingdom	12659	0.74	0.88	35	494	95	**0.13**	−0.15	0.93	**0.45**	**0.36**	0.06	0.07	3.33	**0.16**		
Massachusetts	1723				514	98	**0.1**	−0.25	1.04	**0.35**	**0.26**	0.08	0.17	3.28	0.01		
United States	4978	0.74	0.94	40	481	90	0.05	−0.10	1.06	**0.18**	**0.14**	0.11	0.23	3.28	0.04		
Vietnam	4959	0.69	0.62	70	511	86	**0.12**	0.21	0.63	**0.18**	**0.19**	0.03	0.06	3.08	−0.04		

### Design and statistical analyses

Each participating student spent 2 hours on the PISA survey, including tests measuring reading comprehension, mathematics skills, and science literacy. The PISA design used different sets of problems for different students (rotated design). PISA provides an extensive manual to guide statistical data analysis [57], which we have followed. Both this manual and the PISA technical report [58] provide thorough explanations of the statistical framework underlying PISA. PISA “implemented complex methodological procedures to ensure reliable population estimates and their respective standard errors” [57], and the manual explains, for example, how these data can be used to compare sex differences reliably (e.g., [57]). Achievement levels for mathematics (as for reading and science) were estimated with a Rasch model. In the student-performance dataset PISA provides, 5 plausible scores are provided for the mathematics performance for each student. The samples are representative of the population, and a weight variable is provided for each student, which we used throughout our analyses.

The PISA databases of 2003 and 2012 provide a standardized variable (*ANXMAT*) expressing mathematics anxiety based on 5 statements: “I often worry that it will be difficult for me in mathematics classes”, “I get very tense when I have to do mathematics homework”, “I get very nervous doing mathematics problems”, “I feel helpless when doing a mathematics problem”, and “I worry that I will get poor grades in mathematics” [59]. Students indicated to what degree they agreed with these statements on a 4-point scale. This variable was not available for all students. For the 2003 PISA data, the availability of a mathematics anxiety score per country ranged between 94.8% and 99.9%, and for the 2012 PISA data between 55.1% and 66.7%.

For each student for which data were available, we calculated “excess mathematics anxiety”, which we define as the component of mathematics anxiety adjusted for the mathematics anxiety expected purely based on performance. The rationale is based on the assumption that mathematics anxiety is partially reflecting performance perceptions. In other words, a student who performs poorly in mathematics is likely to be more worried about mathematics related activities simply because the student is not skilled at mathematics. Our specific interest, though, is the degree to which mathematics anxiety deviates from what would be expected based on performance alone. Given that mathematics scores and mathematics performance are expressed on two different scales, we cannot simply subtract these scores. Therefore, we subtracted standardized scores (i.e., with a mean of 0 and a standard deviation of 1) of mathematics performance and anxiety. For each country, we first standardized mathematics anxiety and mathematics performance. Then, we subtracted these two variables for each student and then once more standardized the subtracted measure for the students of each country separately. Please note that we used this variable *in addition* to the standard PISA mathematics anxiety score in a select number of analyses.

Our calculation of average mathematics scores within countries for boys and girls, as well as the calculation of the statistical significance of sex differences (with *p* < .05) follows the detailed guidelines of the instructions for data analysis provided by PISA (OECD, 2003) and has been reported in detail elsewhere [9]. In accordance with the PISA manual, each analysis involving achievement levels was carried out for each plausible value that were then averaged.

For our analyses of parents with a STEM career in the 2012 PISA (the level of needed detail was not available in the 2003 PISA dataset), we classified 65 of the 586 occupation names as “definitely a STEM occupation” across a range of education levels (e.g., software developer, civil engineer, motor vehicle mechanics and repairers, SOM). Our classification is conservative; that is, the occupation clearly relies on the explicit use of mathematics, and while there are occupations that might be classified as STEM by others (e.g., “locomotive engine driver”), our list contained only professions that we felt outside observers would be very likely to also classify as mathematics-related. In the interest of generalizability, we also used a second classification in which we removed 38 lower status STEM jobs from our classification (e.g., we removed any job title that had “repairer”, “technician”, and “installer” from the list). Both operationalizations are likely to disproportionately capture STEM jobs in which there are large sex differences in representation, favoring men. This is because, even within STEM fields, sex differences in interests show significant variation: male-female differences are largest in engineering, physics, and mathematics, and smaller (or even reversed) in medicine and the social sciences [60].

A unique and valuable feature of the 2012 PISA data set is information about how important the parents of each participating child found mathematics (i.e., the earlier 2003 PISA does not contain this information). Parents’ perceived and actual valuation of mathematics were calculated using the associated PISA items. For perceived valuation, we used the mean of two items that asked students to rate how much they agreed with statements about how important their parents find mathematics (“Parents believe studying mathematics is important” and “Parents believe mathematics is important for career”). The students responded on a 4-point scale from strongly agree to strongly disagree. Parents’ actual valuation of mathematics was based on the PISA variable *PQMIMP*, which is a standardized variable (i.e., mean of 0 and standard deviation of 1) based on the following four 4-point scale items (ranging from strongly agree to strongly disagree): 1) “It is important to have good mathematics knowledge and skills in order to get any good job in today’s world”; 2) “Employers generally appreciate strong mathematics knowledge and skills among their employees”; 3) “Most jobs today require some mathematics knowledge and skills”; 4) “It is an advantage in the job market to have good mathematics knowledge and skills”. This latter variable was obtained in only 11 countries and regions (Belgium, Chile, Croatia, Germany, Hong Kong, Hungary, Italy, Macao, Mexico, Portugal, and South Korea). In these countries/regions, there were a total of 100,541 students whose parents completed the parental questionnaire.

Both the students’ beliefs about parental valuation and actual parental valuation are reverse coded. The rationale for the reverse coding was that the aforementioned 4-point scale ran from “strongly agree” (1 point) to “strongly disagree” (4 points), which means that participants with a higher positive valuation of mathematics actually had lower scores. The reverse coding makes the scores more intuitive, such that the calculated scales run from low to high agreement about the importance of mathematics.

We compared single-sex and mixed-sex settings using PISA’s indication of the percentage of girls in a school. We defined single-sex schools as those with 0 or 100% girls and mixed-sex schools as those with a sex ratio of at least ⅓ of the number of students from the lesser-represented sex to the number of students to the greater-represented sex.

In our data analyses, we use an alpha criterion of 5% (i.e., *p* < .05). Further, we report effect sizes as Cohen’s *d* [61]. Cohen’s *d* is defined as the difference of two means divided by the pooled standard variance. Cohen’s *d* is used to express sex differences as a proportion of a standard deviation.

We used the statistical software R for all our analyses [62]. We used the *R* packages “lme4” [63] and “lmerTest” [64] for the reported random intercept model.

### Ethical approval

No institutional ethical approval was necessary for carrying out this secondary data analysis of the publicly available and fully anonymized PISA datasets. It should be noted that parental permission for student participation in the PISA surveys was secured by the staff coordinating PISA data collection, if required by the school or education system [59].

## Results

### Mathematics anxiety as a function of human development

**Fig 1:**
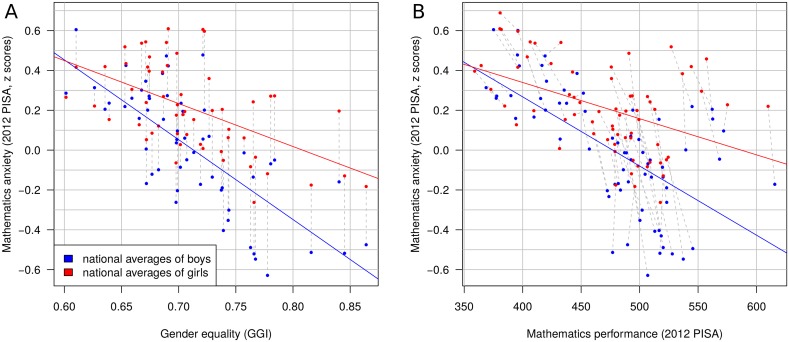
Mathematics anxiety (y-axis) as a function of gender equality (A) and mathematics performance (B) for different countries and economic regions in the 2012 PISA data set. Each data point represents a national average for girls (red) and boys (blue); grey lines connect the two averages of each country. A: Although average national levels of mathematics anxiety were lower in more gender equal countries, the sex differences in mathematics anxiety were larger. B: Consistent with studies of individuals, average national levels of mathematics anxiety were strongly related to average national levels of performance, with higher performing countries having lower levels of mathematics anxiety. This effect was stronger for boys than for girls.

Our first analysis tests if it is indeed the case that higher levels of gender equality and general development are associated with higher levels of mathematics anxiety (all national averages of the 2003 PISA survey are listed in Table 1, and those for the 2012 PISA in Table 2). We used the level of gender equality as measured by the GGI as a proxy of the degree to which basic needs are fulfilled, as well as the more direct measure of need satisfaction, the HDI. In contrast to the hypothesis, both indices were negatively correlated with national averages of mathematics anxiety (see Tables 3 and 4 for all correlations of the 2003 and 2012 variables, respectively). In other words, the national average (i.e., combined score of boys and girls) of mathematics anxiety was lower in more gender equal and developed nations: For the 2003 dataset, the correlation between GGI and mathematics anxiety was *r*(35) = -.75, *p* < .001, and between HDI and mathematics anxiety was *r*(36) = -.62, *p* < .001. For the 2012 dataset, the correlation between GGI and mathematics anxiety was *r*(55) = -.68, *p* < .001 (Fig 1A), and for HDI and mathematics anxiety, *r*(59) = -.61, *p* < .001.

**Table 3:** Pearson correlations between variables listed in Table 1.

	GGI	HDI	PD	math	mathD	anx	anxD
GGI							
HDI	0.57***						
PD	−0.55***	−0.61***					
math	0.52**	0.82***	−0.49**				
mathD	−0.39*	−0.02	−0.03	0.06			
anx	−0.76***	−0.63***	0.68***	−0.63***	0.04		
anxD	0.46**	0.56***	−0.62***	0.44**	0.35*	−0.48**	
EanxD	0.07	0.39*	−0.49**	0.32*	0.73***	−0.29	0.89***

*<.05;

**<.01;

***<.001.

**Table 4:** Pearson correlations between variables listed in Table 2.

	GGI	HDI	PD	math	mathD	anx	anxD	EanxD	ratio1	ratio2	pod
GGI											
HDI	0.54***										
PD	−0.59***	−0.55***									
math	0.45***	0.71***	−0.29*								
mathD	0.04	0.14	−0.23	0.05							
anx	−0.68***	−0.61***	0.58***	−0.56***	0.01						
anxD	0.61***	0.63***	−0.62***	0.58***	0.55***	−0.46***					
EanxD	0.42**	0.48***	−0.53***	0.39**	0.83***	−0.29*	0.91***				
ratio1	0.30*	0.27*	0.03	0.33**	−0.20	−0.27*	0.06	−0.07			
ratio2	0.26	0.13	−0.05	0.08	−0.17	−0.19	−0.10	−0.12	0.67***		
pod	0.23	0.50***	−0.27	0.32**	0.26*	−0.39**	0.37**	0.37**	0.42***	0.35**	
pid	0.58	0.75*	−0.54	0.67*	−0.51	−0.67*	0.33	0.01	−0.14	−0.10	0.33

*<.05;

**<.01;

***<.001.

The above results, however, are potentially confounded by the relation between mathematics anxiety and mathematics performance, which we address below. Overall, national levels of mathematics anxiety were *lower* in countries with *higher* national performance levels in both the 2003 (*r*[39] = -.63, *p* < .001) and 2012 (*r*[66] = -.56, *p* < .001, Fig 1B) assessments. In both PISA surveys, this effect was stronger for the national averages of boys than for those of girls, as determined by the interaction between mathematics performance and sex using a random intercept model, with individuals nested within countries (for 2012: *β* = -0.0017, *p* < .001, for 2003: *β* = -0.0012, *p* = .001). Thus, as for the relation between gender equality and mathematics anxiety, overall levels of mathematics anxiety are lower in the higher performing countries, but the sex difference in mathematics anxiety in those countries is larger.

### Mathematics anxiety controlled for performance

Importantly, the sex difference in mathematics performance differed from the sex difference in mathematics anxiety: The overall sex difference in mathematics anxiety was small to moderate in size (average effect size, Cohen’s *d*, was 0.28 in 2003 and 0.23 in 2012) and girls were found to have a significantly higher level of mathematics anxiety in the majority of countries (95% in 2003, and 82% in 2012). This sex difference in mathematics anxiety was more than twice the magnitude of the sex difference in mathematics performance (average effect size, Cohen’s *d*, was 0.11 in 2003 and 0.09 in 2012, see Tables 1 and 2). Girls were found to score significantly lower than boys in mathematics performance in 66% of countries in 2003 and 59% in 2012. In other words, in nearly all countries, girls reported higher levels of mathematics anxiety than expected based on performance alone. We will address this trend next.

To control for the relation between mathematics performance and mathematics anxiety and to better capture the sex differences in these areas, we calculated for each student the standardized mathematics anxiety score minus the standardized mathematics performance score on a country-by-country basis, as noted above. The difference is a measure of ‘excess’ mathematics anxiety beyond what would be expected based on performance (see Materials and Methods for exact calculation). For both PISA assessments, girls exhibited more ‘excess’ mathematics anxiety than did boys (2003: *d* = 0.25; 2012: *d* = 0.21). For the 2012 PISA, the sex difference in excess anxiety correlated with the GGI, *r*(55) = .43, *p* < .001, and with the HDI, *r*(59) = .48, *p* < .001. For the 2003 PISA data, only the correlation between the sex difference in excess anxiety and HDI was significant, *r*(36) = .40, *p* = .013 (the correlation with GGI was *r*[35] = .06, *p* = .740). In the following analyses, we will use both the national averages of regular mathematics anxiety and the excess anxiety scores.

### Power distance and single-sex schooling

One explanation for the larger sex differences in mathematics anxiety in more gender-equal countries was that these countries had a smaller power distance, leading to more between-sex comparisons (as suggested by proponents of the gender stratification model, see Introduction). Power distance was negatively correlated with gender equality, *r*(49) = -.61, *p* < .001, and levels of human development, *r*(50) = -.56, *p* < .001 (data of 2012). In other words, countries with a lower level of power distance had a higher level of gender equality and higher general economic and social development. Because we found that gender equality is related to sex differences in mathematics anxiety, there may be a relation between power distance and the sex difference in mathematics anxiety as well. We first tested if power distance was indeed correlated with the sex difference in mathematics anxiety and found that is was. We found this to be the case for both the 2003, *r*(35) = -.68, *p* < .001, and the 2012 dataset, *r*(51) = -.66, *p* < .001. This was also the case for the performance adjusted excess mathematics anxiety in both 2003, *r*(35) = -.49, *p* = .002, and 2012, *r*(51) = -.54, *p* < .001. In other words, countries with a smaller power distance have a larger sex difference in mathematics anxiety.

A more direct prediction of the power-distance hypothesis, though, was that the opportunity for and the degree of between-sex comparisons contributes to the sex differences in mathematics anxiety observed across countries. To test this feature of the power distance hypothesis, we compared boys and girls in single-sex and mixed-sex schools (for both the 2003 and 2012 PISA). To do so, we first calculated for each country the average level of mathematics anxiety of students in single-sex and mixed-sex schools for both boys and girls. Next, we applied a within-country analysis of variance with the factors sex (boys vs. girls) and school type (single vs mixed sex). Consistent with the above analyses, in the 2012 PISA girls had higher mathematics anxiety than boys, *F*(1,53) = 107.6, *p* < .001, and there was a trend for school type, *F*(1,53) = 2.93, *p* = .092, with students in single sex schools scoring slightly higher on mathematics anxiety. For 2003, we also found similar main effect of sex, *F*(1,17) = 54.68, *p* < .001, and the opposite effect of school type, *F*(1,17) = 17.4, *p* < .001, with students in mixed-sex schools scoring higher on mathematics anxiety. It should be noted that the number of countries of the 2003 data set for which mathematics anxiety data was available for both single-sex and mixed-sex schools was limited (only 18 out of 41 countries). Critically, there was no interaction between sex and school-type in 2012, *F*(1,53) = 2.20, *p* = .144, or 2003, *F*(1,17) = 0.85, *p* = .382; the effect of school type and its interaction with sex were also not significant when performance adjusted excess mathematics anxiety was used as the outcome measure. These results indicate the sex difference in mathematics anxiety is not related to the frequency of between-sex interactions, at least in school settings.

### Mathematics anxiety and the role of parents

Thus far, we have shown that overall (i.e., the total score of both boys and girls) mathematics anxiety is lower in countries with higher levels of gender equality and human development, whether or not mathematics performance is statistically controlled. Further, the sex difference in mathematics anxiety did not differ between students in same-sex and mixed-sex schools.

These findings are inconsistent with predictions based on the gender stratification hypothesis. Still, it is possible that socio-cultural patterns in occupational choices (e.g., few or many mothers working in STEM) and the (potentially associated) parental valuation of mathematics for sons compared to daughters contributed to observed sex differences in mathematics anxiety in ways consistent with gender stratification. We assessed these possibilities in three ways, namely by examining the relation between mathematics anxiety and (1) the ratio of mothers to fathers working in STEM fields in each country’s sample, (2) boys’ and girls’ reports about their parents valuation of mathematics, and (3) parents’ beliefs about the importance of mathematics for their sons and daughters.

First, the ratio of mothers to fathers working in STEM jobs varied considerably among countries and regions (from 0.02 in Peru to 0.33 in Shanghai), and as expected, the ratio of mothers in STEM was higher in more gender equal and in more developed countries, GGI, *r*(54) = .29, *p* = .026, and HDI, *r*(58) = .28, *p* = .029. Critically, this ratio was neither correlated with the sex difference in mathematics anxiety, *r*(65) = .06, *p* = .611, nor the sex difference in mathematics performance, *r*(65) = -.18, *p* = .143. Next, we tested for the robustness of our results in relation to our classifications of STEM jobs by including only higher status STEM occupations, for the reason that the desirability of a job is not only influenced by the the nature of the work but also the social status that accompanies that position. Importantly, this distinction did not influence the results: When we excluded STEM jobs which included terms such as “repairer” or “operator” and only included engineers, architects, designers, and developers, we found similar relations. This ratio of mothers to fathers with a high-status STEM job was neither correlated with the sex difference in mathematics anxiety, *r*(65) = -.08, *p* = .498, nor the sex difference in mathematics performance, *r*(65) = -.168, *p* = .175.

**Fig 2:**
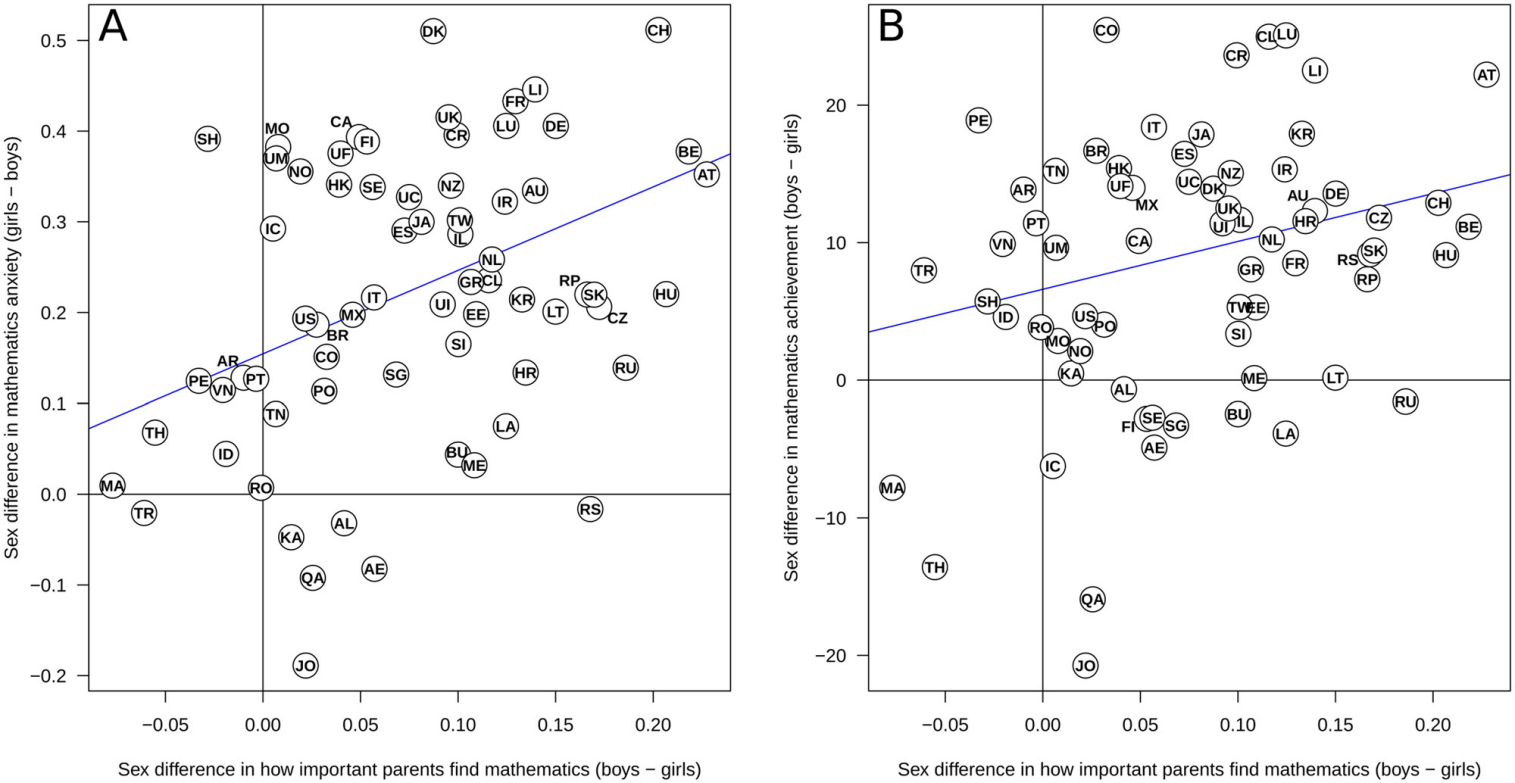
Sex differences in parental opinion (as reported by the students) about the importance of mathematics are related to sex differences in mathematics anxiety and performance. A: The larger the difference in boys’ and girls’ view of parental valuation of mathematics for boys and girls, the larger the sex differences in mathematics anxiety. B: The larger the difference in parental valuation of mathematics for boys and girls, the larger the sex differences in mathematics performance. For abbreviations, see Materials and Methods. Notes: AE: United Arab Emirates, AL: Albania, AR: Argentina, AT: Austria, AU: Australia, BE: Belgium, BR: Brazil, BU: Bulgaria, CA: Canada, CH: Switzerland, CL: Chile, CO: Colombia, CR: Costa Rica, CZ: Czech Republic, DE: Germany, DK: Denmark, EE: Estonia, ES: Spain, FI: Finland, FR: France, GR: Greece, HK: Hong Kong, HR: Croatia, HU: Hungary, IC: Iceland, ID: Indonesia, IL: Israel, IR: Ireland, IT: Italy, JA: Japan, JO: Jordan, KA: Kazakhstan, KR: South Korea, LA: Latvia, LI: Liechtenstein, LT: Lithuania, LU: Luxembourg, MA: Malaysia, ME: Montenegro, MO: Macao, MX: Mexico, NL: The Netherlands, NO: Norway, NZ: New Zealand, PE: Peru, PO: Poland, PT: Portugal, QA: Qatar, RO: Romania, RP: Perm (Russia), RS: Serbia, RU: Russia, SE: Sweden, SG: Singapore, SH: Shanghai, SI: Slovenia, SK: Slovak Republic, TH: Thailand, TN: Tunisia, TR: Turkey, TW: Chinese Taipei, UI: Uruguay, UC: US state Connecticut, UF: US state Florida, UK: United Kingdom, UM: US state Massachusetts, US: United States, VN: Vietnam.

Second, across countries, girls reported that parents find mathematics less important than boys did, *d* = 0.11, *t*(67) = 8.79, *p* < .001. The degree to which boys and girls differed in this was related to the sex difference in mathematics anxiety (Fig 2A), *r*(66) = .36, *p* = .002, excess mathematics anxiety, *r*(66) = .35, *p* = .003, and to the sex difference in mathematics performance (Fig 2B), *r*(66) = .30, *p* = .013. Together, these findings provide convergent reliability for the cross-national measurement of sex differences in mathematics anxiety and indicate it is correlated with students’ perceptions of parental valuation of mathematics.

**Fig 3:**
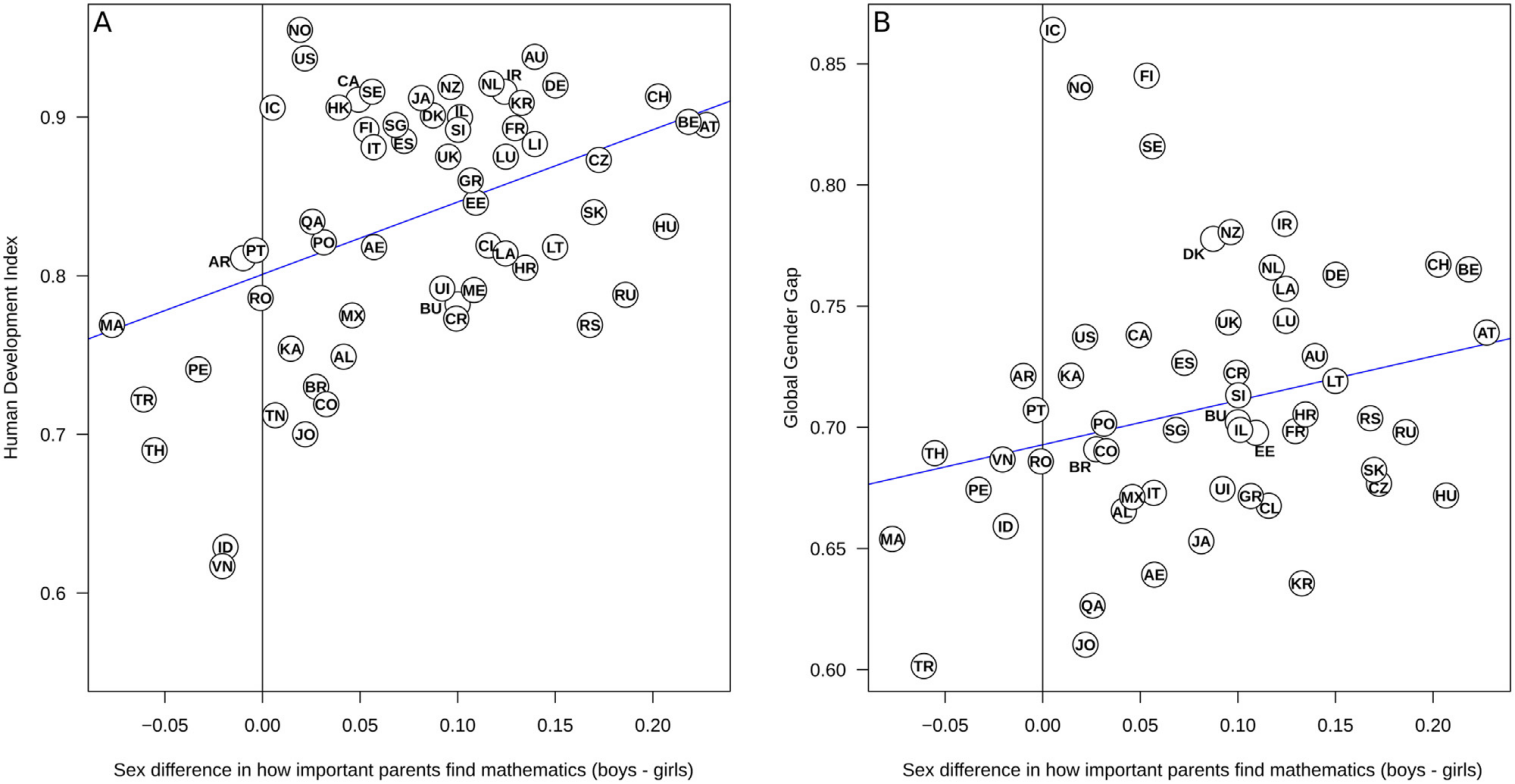
Sex differences in parental opinion (as reported by the 15-year olds) about the importance of mathematics are related to human development (HDI) and gender equality (GGI). The two-letter codes are country abbreviations (see SOM). A: The sex difference in reported parental valuation of mathematics was larger in more developed countries. B: A similar effect was found for gender equality, although the Nordic countries Iceland (IC), Finland (FI), Sweden (SE) and Norway (NO) appear to deviate from the international trend. Notes: AE: United Arab Emirates, AL: Albania, AR: Argentina, AT: Austria, AU: Australia, BE: Belgium, BR: Brazil, BU: Bulgaria, CA: Canada, CH: Switzerland, CL: Chile, CO: Colombia, CR: Costa Rica, CZ: Czech Republic, DE: Germany, DK: Denmark, EE: Estonia, ES: Spain, FI: Finland, FR: France, GR: Greece, HK: Hong Kong, HR: Croatia, HU: Hungary, IC: Iceland, ID: Indonesia, IL: Israel, IR: Ireland, IT: Italy, JA: Japan, JO: Jordan, KA: Kazakhstan, KR: South Korea, LA: Latvia, LI: Liechtenstein, LT: Lithuania, LU: Luxembourg, MA: Malaysia, ME: Montenegro, MO: Macao, MX: Mexico, NL: The Netherlands, NO: Norway, NZ: New Zealand, PE: Peru, PO: Poland, PT: Portugal, QA: Qatar, RO: Romania, RP: Perm (Russia), RS: Serbia, RU: Russia, SE: Sweden, SG: Singapore, SH: Shanghai, SI: Slovenia, SK: Slovak Republic, TH: Thailand, TN: Tunisia, TR: Turkey, TW: Chinese Taipei, UI: Uruguay, UC: US state Connecticut, UF: US state Florida, UK: United Kingdom, UM: US state Massachusetts, US: United States, VN: Vietnam.

Further, the sex difference between boys’ and girls’ reports of how important their parents view mathematics was larger in more developed countries (correlation with HDI, *r*[59] = .48, *p* < .001, Fig 3A). The relation between parental views and gender equality (GGI) is more difficult to interpret, because a visual inspection of the scatter plot suggests that the Nordic countries seem to form a separate cluster (Fig 3B). We calculated the Cook’s Distance (commonly defined as 4[*N*-*K*-1]) of these points and found that Norway and Iceland were well beyond the commonly used Cook’s Distance cut-off point. When these two outliers are excluded, the correlation was statistically significant, *r*(53) = 0.33, *p* = .014 (see Discussion for further comments on this interpretation).

Although the actual parental evaluations of the importance of mathematics was available in only 11 countries, we also found in this small subset of countries that parents of girls reported that mathematics was less important than did parents of boys, *d* = 0.1, paired *t*(10) = 4.7, *p* < .001. This result is consistent with boys’ and girls’ reports of parental valuation of mathematics for the larger sample of countries.

## Discussion

We examined sex differences in mathematics anxiety and related variables among adolescents to test predictions following from the gender stratification hypothesis, and in doing so provide several novel insights into the nature and social correlates of the sex difference in mathematics anxiety and performance.

### Summary of findings and interpretation

First, our results confirmed that mathematics anxiety is higher in girls than in boys. Novel in our approach, though, was that we accounted for the level of mathematics performance that might explain sex differences in mathematics anxiety, and demonstrated a consistent, across-country, ‘excess’ in girls’ mathematics anxiety.

Importantly, contra predictions of the gender stratification hypothesis, our results unequivocally demonstrated that mathematics anxiety cannot be considered a ‘luxury’ problem that only manifests in gender equal countries with higher levels of human development. This is because the overall levels of mathematics anxiety of both sexes are, in contrast to an earlier claim [15], lower in more gender equal and more developed countries.

Further, we clarified the previously reported exaggeration of the sex difference in mathematics anxiety in more gender-equal countries. We showed that there are countries in which both girls and boys have a high level of mathematics anxiety, but with no sex differences in anxiety. Indeed, overall mathematics anxiety levels and sex differences in mathematics anxiety appear to be two different, albeit related phenomena. We showed that economically developed and more gender equal countries have a *lower overall level* of mathematics anxiety, and yet a *larger sex difference* in mathematics anxiety relative to less developed countries. Our results point to a more complex pattern whereby the mean level of mathematics anxiety decreases with development, but more so for boys than for girls. This will have important implications for policy makers, as we discuss below.

We addressed another explanation, derived from the gender stratification hypothesis, for the finding that sex differences in mathematics anxiety are larger in more gender equal countries: that fewer between-sex comparisons are made in less gender equal countries, due to the larger power distance in these societies. We showed that power distance is indeed correlated with the sex difference in mathematics anxiety (i.e., countries with a smaller power distance have a larger sex difference in mathematics anxiety), although this variable is also correlated with gender equality and human development; countries with high levels of power distance are often less-developed and less gender equal countries. Instead of using the proxy variable power distance, we took a more direct route of testing the hypothesis by using information on single-sex versus mixed-sex schooling provided in the PISA data set. We reasoned that if the likelihood or frequency of between-sex comparisons plays a role (as suggested by proponents of the gender stratification model), we should observe different patterns of sex differences in mathematics anxiety among children in single-sex and mixed-sex schools. However, inconsistent with the hypothesis, we found no relation between sex differences in mathematics anxiety and attending a mixed-sex or single-sex school.

We did find a non-significant trend of higher mathematics anxiety in single-sex schools (across both boys and girls) in 2012 and significantly higher mathematics anxiety in mixed-sex schools in 2003. We believe that caution needs to be exercised in the interpretation of these results; importantly, it is irrelevant for the main focus of the current study. It is unclear whether this reflects an effect of selection of different types of students into mixed- vs. same-sex schools [65], an emergent social phenomenon in schools composed of mixed- or same-sex students, or an effect of other school characteristics on children’s mathematics anxiety. This point, though, goes well beyond the topic of the current study, and should be addressed in a separate study.

Our study also expanded the assessment of potential socio-cultural influences on sex differences in mathematics anxiety, with the inclusion of parents’ occupations, and parents’ perceived and actual valuation of the mathematical development of their children. We found that the proportion of mothers to fathers in STEM occupations was unrelated to mathematics performance or mathematics anxiety. This contrasts with an earlier interpretation of labor statistics of women in research [15]. In essence, when only considering the occupation of parents, there is no reason to assume that children’s mathematics performance or anxiety is influenced by parental occupation in the STEM sector. It is, in principle, possible that female role models can have both positive effects on girls’ mathematics related affect by breaking down stereotypes [3] but also negative effects on girls’ mathematics related affect, as found by others [66, 67] in studies of schoolgirls engaging with STEM role models. One interpretation of these studies is that the positive and negative effects cancel each other out. Although the latter interpretation is speculative, there is no strong case to make that same-sex role models would make a substantive difference in the under-representation of women in STEM occupations [68], despite many social commentators saying that it will. At the very least, we need a better understanding of the influence of the social environment before effective interventions based on role models for girls can be recommended.

Further, we found that boys reported higher perceived parental valuation of mathematics than did girls, and parents actually rated mathematical development as more important for sons than for daughters. The differential valuation of mathematics between the sexes was larger in more developed countries. Paradoxically, economic and social development was associated with a widening gap between parents’ beliefs about the importance of mathematics for sons versus daughters. We found that in this respect the Nordic countries differ from most other countries: These countries score highest in gender equality and have no (Iceland) or a small (Norway, Sweden, Finland) difference between parental valuation of sons’ and daughters’ mathematical development. Further study of the unique socio-cultural factors affecting sex differences in these specific countries will be of importance for better understanding the relation between gender equality and gender differences in educational attitudes.

### A proposal for an alternative explanation of the findings

Our findings do not provide support for a number of key predictions of the gender-stratification hypothesis, in particular the larger sex difference in mathematics anxiety in more gender equal countries and the finding that the difference in parental valuation for boys and girls is generally largest in highly gender equal countries (except for the Scandinavian countries). As such, we propose an alternative explanation. Before doing so, we would like to make two more general points. First, our rejection of the gender stratification hypothesis is not a general rejection of the idea that socio-cultural factors may play an important role explaining sex differences in mathematics anxiety and more general in educational and occupational choices. In fact, there are a number of alternative models that focus on the socio-cultural factors (e.g., [69–71]), and our current data set does not further address the details of such models. Our main point is that there is a lack of evidence to support the assumption that economic or educational inequities are responsible for today’s observed sex differences in mathematics affect, attitudes, and performance. Second, our main conclusion is that the development of an alternative explanation to the influential gender-stratification model is therefore preferable; below, we make a suggestion of what such an alternative may look like, but this alternative model goes well beyond the main aim of our study and beyond the dataset.

Our findings are consistent with other recent findings of larger sex differences in these countries across many cognitive abilities (e.g., spatial ability [72]), behavioral expressions (e.g., crying [73]), major personality traits [73], and even biological traits (e.g., height, [73]). Broadly, as conditions associated with human development (e.g., health) and gender-equality (e.g., women’s participation in politics) improve, people’s opportunities in many domains of life increase and individual and sex differences in many traits increase with them. With respect to STEM, it is possible that parents and students with less economic hardship consider a wider array of career options.

We propose that while economic considerations may play a more prominent role in STEM-related interest for individuals living in less developed countries, intrinsic subject-specific interest will play a more important role in educational and occupational attitudes and choices for individuals living in countries with higher levels of economic well-being. When the relative role of interests become more important than the financial drivers, and when men and women have more freedom to pursue their intrinsic interests, the well established sex difference in occupational interests will become more strongly expressed [74–77]. Altogether, these patterns might explain why girls benefit less than boys in terms of reduced mathematics anxiety. For example, in more developed countries in which people engage more in activities that intrinsically interest them, girls might not engage in STEM activities as much as boys, giving them less opportunity to reduce their negative feelings about mathematics.

Further, we propose that the influence of parental opinion on children’s mathematics anxiety is not well-established, and that correlations may reflect the influence of children’s interests on their parents’ opinions at least as much as parents influence their children’s interests. We hypothesize that when parents are asked by researchers how important they value mathematics for the future of their children, parents will likely take into account the levels of mathematics-related anxiety and interest in mathematics that children express at home and with respect to their mathematics experiences in school. Consistent with this hypothesis, children’s attitudes toward learning predict changes in their parents’ educational expectations starting even before the school years [78]. Because girls express higher levels of anxiety about mathematics, parents of girls may be more likely to devalue the importance of the domain in relation to their daughters than their sons, who show relatively less mathematics anxiety by comparison.

It will be difficult to directly test the latter proposal empirically. We are aware of one study, though, that demonstrates that shared environmental factors (environmental factors that make siblings more similar to each other) do not explain substantial variation in levels of mathematics anxiety [79]. If parental opinions regarding the importance of mathematics have a major effect on mathematics anxiety, we would expect a larger shared environment effect. Instead, Wang and colleagues [79] argue that genetic and child-specific parent-child interactions may play an important explanatory role. We believe that it is too early to draw strong conclusions from this research, but that there are good reasons to be skeptical of the idea that the general role modeling and opinions expressed by parents are the most potent influences on individual differences in children’s mathematics anxiety.

### Limitations

One important limitation of PISA data is that they are correlational in nature; it is impossible to draw conclusions about causal pathways, even though some are more likely than others. One of the difficulties with the relation between self-concept variables and performance is that it is not clear which influences which, and how much they interact with one another. Our approach of subtracting performance and anxiety scores helps, but ultimately, longitudinal and experimental studies are needed to better understand the causal pathways.

Readers may at this point argue that we have only analyzed one specific affect variable (i.e., mathematics anxiety), while there are other important self-concept variables, such as mathematics self concept and mathematics efficacy. In fact, we also analyzed these two variables in the same way as mathematics anxiety and found similar data patterns (see SOM for details). We believe, however, that mathematics anxiety is the most important variable, because of its well established role in forming a barrier in keeping students from mathematics. Moreover, its distinct neural signature indicating a learned fear response provides a critical validation of the concept and reveals that it is a complex phenomenon that can manifest as a type of acquired phobia centered on mathematics.

### Outstanding questions

Our data analyses not only help to evaluate implications from the influential gender-stratification model. Our data also raise new questions, naturally stemming from limitations in the data or in our methodology. An outstanding question is the degree to which the PISA measure captures mathematics anxiety. For example, Bieg and colleagues [2] measured different types of mathematics anxiety, namely state (i.e., “in the moment”) and trait anxiety (which is the type measured in the PISA surveys). They found that in their sample of German 9th and 10th graders girls had a higher level of trait anxiety, but there were no sex differences in state anxiety. Interestingly, the authors found that the degree to which girls subscribed to gender stereotypes had a higher level of trait mathematics anxiety relative to their state mathematics anxiety. It would be of great interest to determine if this relation between subscribing to gender stereotypes and mathematics anxiety is also observed in other countries, and also what the exact causal relation between these variables is.

### Conclusions for policy makers

The findings and conclusions of the current study are not only relevant from a theoretical academic perspective, but also highlight challenges for policy makers to simultaneously increase mathematics performance (c.f., [80]) and ensure equal opportunities for STEM participation, yet also ensure that girls and women will not avoid mathematics more than boys and men due to mathematics anxiety. Policies must take into consideration that sex differences in career choices are not a simple function of gender equality and equal opportunities; and that, paradoxically, other factors (e.g., sex differences in occupational interests [74–76] and sex differences in other skills [9, 81, 82]) emerge in highly developed, gender-equal countries that might disproportionately affect girls’ mathematics anxiety and participation in STEM.

Finally, the finding of larger sex differences in mathematics anxiety in countries with a larger proportion of mothers to fathers in STEM occupations may be viewed negatively, but it may equally well be viewed as positive. After all, it shows that despite a larger sex difference in mathematics anxiety in these countries, this does not preclude a relatively high proportion of mothers choosing a career in STEM subjects. Indeed, actual mathematical competence, which is relatively high overall for both sexes in most of these countries, is likely a more critical trait for STEM entry than relative mathematics anxiety [83].

## Supporting Information

S1 Supplementary Online Material Supplementary Online Material (SOM). (PDF)
