# Supplementary material for: Countries with Higher Levels of Gender Equality Show Larger National Sex Differences in Mathematics Anxiety and Relatively Lower Parental Mathematics Valuation for Girls
Source: PLoS One. 2016 Apr 21;11(4):e0153857. doi: 10.1371/journal.pone.0153857 (PMC4839696; doi:10.1371/journal.pone.0153857)
Supplement: S1 Supplementary Online Material — (PDF) [file pone.0153857.s001.pdf]

Supplementary Online Material

Content:

1) Analysis of other self-concept variables
2) Details of categorization of parental occupation used in main manuscript

Abbreviations used:
PISA: Programme for International Student Assessment
GGI: Global Gender Gap Index
HDI: Human Development Index

Analysis of other self-concept variables

The variables and samples: In the main manuscript, we focus on the mathematics anxiety (PISA variable "ANXMAT"). However, we carried out the same analyses as in the main manuscript on the self-concept variables "mathematics self concept" (PISA variable "*SCMAT*") and "mathematics efficacy" (PISA variable "*MATHEFF*"). The analyses revealed a similar relation between the socio-cultural factors and these different (but related) self-concept variables as found for mathematics anxiety (main text).

Both the PISA databases of 2003 and 2012 provide the standardized variable MATHEFF expressing mathematics self-efficacy based on the following 8 statements (OECD, 2014, p.322).

- Using a train timetable to work out how long it would take to get from one place to another
- Calculating how much cheaper a TV would be after a 30% discount
- Calculating how many square metres of tiles you need to cover a floor
- Understanding graphs presented in newspapers
- Solving an equation like 3x+5= 17
- Finding the actual distance between two places on a map with a 1:10 000 scale
- Solving an equation like 2(x+3) = (x + 3) (x - 3)
- Calculating the petrol consumption rate of a car

Students indicated to what degree they agreed with these statements on a 4-point scale. This variable was not available for all students. For the 2003 PISA data, the availability of a mathematics self efficacy score per country ranged between 95% and 100%, and for the 2012 PISA data between 55% and 67%.

The PISA databases of 2003 and 2012 provide a standardized variable SCMAT expressing mathematics self-concept based on 5 statements (OECD, 2014, p.323).

- I am just not good at mathematics
- I get good in mathematics
- I learn mathematics quickly
- I have always believed that mathematics is one of my best subjects
- In my mathematics class, I understand even the most difficult work

Students indicated to what degree they agreed with these statements on a 4-point scale. This variable was not available for all students. For the 2003 PISA data, the availability of a mathematics self concept score per country ranged between 95% and 100%, and for the 2012 PISA data between 55% and 67%.

Results:

Correlations of national averages
National averages of self efficacy correlate with HDI (for 2003 PISA, $r(36)=.40$, $p=.01$; for 2012 PISA data, $r(59)=.45$, $p<.001$. Thus, higher levels of development are associated with higher national averages of mathematics efficacy. However, the correlation between self efficacy with GGI is weak 2003, $r(35)=.32$, $p=.057$, and was not observed in 2012, $r(55)=.21$, $p=.11$.

National averages of self concept did not correlate with HDI in 2003, $r(36)=-.08$,$p=.64$ or 2012, r(59)=-.09, $p<.49$. Similarly, self concept did not correlate with GGI in 2003, r(35)=.22,p=.19, or 2012, $r(55)=.06$,$p=.66$.

Thus, while national averages of mathematics anxiety relate strongly to HDI and GGI (the higher the level of development, the lower the national level of mathematics anxiety), the relation is weaker for self efficacy and not found for self concept.

Relation between self efficacy and mathematics performance

There was a positive correlation between mathematics performance and self efficacy in 2003, $r(39)=.45$,$p=.003$, and in 2012, $r(66)=.60$, $p<.001$.

The correlation between mathematics performance and self concept was weaker in 2003, $r(39)=-.26$,$p=.09$, and in 2012, $r(59)=-.32$,$p=.006$.

Thus, national averages of self efficacy are positively correlated with math performance, whereas the opposite is true for self concept. The finding is that higher levels of mathematics performance are associated with lower levels of self concept. This is not a new finding, though (Stevenson et al., 1990). When looking at the self-concept questions (above), it is obvious that the better schools and classes are, the less likely students will answer, for example, that they even understand the most difficult work.

Sex differences in mathematics efficacy and self concept

Overall levels of mathematics efficacy were higher in countries with higher mathematics performance. A random intercept model (similar to that in the main manuscript) shows that this relation was stronger for boys than for girls (for 2012: β=-0.0011,p<.001, for 2003: β=-0.0013,p<.001). Further, overall levels of self concept were lower in countries with higher mathematics performance. Here too, the random intercept model shows that this effect was stronger for boys than for girls (for 2012: β=0.0014, p<.001, for 2003: β=-0.0015,p<.001).

Similar to mathematics anxiety, there were significant sex differences (boys scoring higher) in both mathematics efficacy (international average in Cohen's d in 2003: 0.34 and 0.28 in 2012) and mathematics self concept (international average in Cohen's d in 2003: 0.33 and 0.30 in 2012) .

Further, the sex differences in mathematics efficacy correlated with GGI (2003: $r(35)$=.37,p=.02; 2012: $r(55)$=.52, p<0.01), and with HDI (2003: $r(36)$=.59,p<.001; 2012: $r(59)$=.74, p<.001). And similarly, the sex differences in self concept correlated with GGI (2003: $r(35)$=.40,p=.01; 2012: $r(55)$=.49, p<0.01), and with HDI (2003: $r(36)$=.55,p<.001; 2012: $r(59)$=.63, p<.001).

In the main paper, we adjusted mathematics anxiety for mathematics performance by subtracting the normalized scores of both variables for each student. We carried out the same calculation for mathematics efficacy and self concept. Following this calculation, we found the adjusted international average sex difference in self efficacy was 0.23 sd in 2003 and 0.18 in 2012. Similarly, the adjusted international average sex difference in mathematics self concept in 2003 was 0.20 sd and 0.18 sd in 2012.

Correlations between sex differences in adjusted scores, GGI and HDI

When mathematics efficacy is adjusted for mathematics performance, it again correlates positively with HDI (2003: $r(36)=.53$, $p<.001$; 2012: $r(59)=.56, p<.001$) and with GGI (2003: $r(35)=.60$, $p<.001$; $r(55)=.53$, $p<.001$). Similarly, performance-adjusted sex differences in mathematics self concept correlated with GGI in 2003, $r(35)=.66$, $p<.001$ and in 2012, $r(55)=.50$, $p<.001$. Performance-adjusted sex differences in mathematics self concept correlated with HDI in 2003, $r(36)=.49$, $p=.001$ and in 2012, $r(59)=0.53, p<.001$.

Power distance

In the main manuscript, we reported that power distance was correlated with national levels of mathematics anxiety. We did not find such a correlation for mathematics efficacy, but we found a link for mathematics self concept in 2012, $r(51)=-.28$, $p=.04$ (see Table S1 for all correlations).

Table S1

	2003	2012
overall math efficacy	$r(35)=-.20, p=.24$	$r(51)=-.17$, $p=.23$
overall math self concept	$r(35)=-.23, p=.18$	$r(51)=-.28$, $p=.04$
sex difference in math efficacy	$r(35)=-0.20, p=0.23$	$r(35)=-0.5$, $p=0.23$
sex difference in math self concept	$r(35)=-0.23, p=.18$	$r(51)=-.28$, $p=.05$

Single sex schooling

Similar to the analysis of variance testing testing the relation between mathematics anxiety and single/mixed sex schooling, the interaction between gender and type of school was not significant for either mathematics efficacy or self concept.

Parent STEM ratio

In the main manuscript, it was shown that the ratio of fathers to mothers with STEM occupations was not correlated with mathematics anxiety. The same was true for

efficacy (STEM: $r(65)=.07, p=.581$; high-status STEM: $r(65)=.0098, p=.937$) and self concept (STEM: $r(65)=.005$, $p=.966$; high-status STEM: $r(65)=-.19, p=.1332$).

Parental valuation

In the main manuscript, it was shown that the larger the sex difference in mathematics anxiety, the larger the sex difference in students' perception of parental valuation. The same effect was found for self efficacy, $r(66)=0.57$, $p<.001$, and for self-concept, $r(66)=0.35, p<.01$. This effect is weaker or non-existent when the performance-adjusted scores are used (mathematics efficacy, $r(66)=0.36, p<.01$; self concept, $r(66)=.10$, $p=.43$).

<u>Details of categorization of parental occupation used in main manuscript</u>

In the main manuscript, we use the proportion of fathers to mothers working in Science, Technology, Engineering, and Mathematics (STEM) occupations. PISA does not categorize occupations as STEM, and we have therefore classified all 586 listed occupations as either "definitely a STEM occupation" and those that are not.

We considered the following 65 as "definitely a STEM occupation":
Agricultural and industrial machinery mechanics and repairers, Air conditioning and refrigeration mechanics, Aircraft engine mechanics and repairers, Aircraft pilots and related associate professionals, Air traffic safety electronics technicians, Applications programmers, Architects, planners, surveyors and designers, Broadcasting and audio-visual technicians, Building and related electricians, Building architects, Chemical and photographic products plant and machine operators, Chemical and physical science technicians, Chemical engineering technicians, Chemical engineers, Chemical processing plant controllers, Chemical products plant and machine operators, Civil engineering technicians, Civil engineers, Computer network and systems technicians, Computer network professionals, Construction managers, Construction supervisors, Electrical engineering technicians, Electrical engineers, Electrical equipment installers and repairers, Electronics and telecommunications installers and repairers, Electronics engineering technicians, Electronics engineers, Electronics mechanics and servicers, Electrotechnology engineers, Engineering professionals (excluding electrotechnology), Engineering professionals not elsewhere classified, Financial and mathematical associate professionals, Geologists and geophysicists, Glass and ceramics plant operators, Industrial and production engineers, Machinery mechanics and repairers, Mathematicians, actuaries and statisticians, Mechanical engineering technicians, Mechanical engineers, Mechanical machinery assemblers, Metal working machine tool

setters and operators, Mineral and stone processing plant operators, Mining and metallurgical technicians, Mining engineers, metallurgists and related professionals, Motor vehicle mechanics and repairers, Petroleum and natural gas refining plant operators, Photographic products machine operators, Physical and earth science professionals, Physical and engineering science technicians, Physical and engineering science technicians not elsewhere classified, Precision-instrument makers and repairers, Science and engineering associate professionals, Science and engineering professionals, Ship and aircraft controllers and technicians, Ships engineers, Software and applications developers and analysts, Software and applications developers and analysts not elsewhere classified, Software developers, Steam engine and boiler operators, Telecommunications and broadcasting technicians, Telecommunications engineering technicians, Telecommunications engineers, Web and multimedia developers, Web technicians.

And the following not as "definitely a STEM occupation":
Actors, Advertising and marketing professionals, Advertising and public relations managers, Aged care services managers, Announcers on radio, television and other media, Apiarists and sericulturists, Archivists and curators, Artistic, cultural and culinary associate professionals, Astrologers, fortune-tellers and related workers, Athletes and sports players, Audiologists and speech therapists, Authors and related writers, Authors, journalists and linguists, Bakers, pastry-cooks and confectionery makers, Bartenders, Beauticians and related workers, Chefs, Child care services managers, Child care workers, Child care workers and teachers aides, Cleaners and helpers, Cleaners and helpers in offices, hotels and other establishments, Cleaning and housekeeping supervisors in offices, hotels and other establishments, Clearing and forwarding agents, Commissioned armed forces officers, Community health workers, Companions and valets, Conference and event planners, Contact centre information clerks, Contact centre salespersons, Cooks, Creative and performing artists, Creative and performing artists not elsewhere classified, Debt-collectors and related workers, Deep-sea fishery workers, Dental assistants and therapists, Dentists, Dieticians and nutritionists, Dispensing opticians, Domestic cleaners and helpers, Domestic, hotel and office cleaners and helpers, Domestic housekeepers, Door to door salespersons, Draughtspersons, Drivers of animal-drawn vehicles and machinery, Early childhood educators, Education managers, Fashion and other models, Fast food preparers, Field crop and vegetable growers, Filing and copying clerks, Film, stage and related directors and producers, Fire-fighters, Fishery and aquaculture labourers, Fishery workers, hunters and trappers, Fitness and recreation instructors and program leaders, Floor layers and tile setters, Food and beverage tasters and graders, Food preparation assistants, Food processing and related trades workers, Food service counter

attendants, Freight handlers, Fruit, vegetable and related preservers, Fumigators and other pest and weed controllers, Garbage and recycling collectors, Garden and horticultural labourers, Gardeners, horticultural and nursery growers, Garment and related pattern-makers and cutters, Garment and related trades workers, Hairdressers, Hairdressers, beauticians and related workers, Health associate professionals, Health associate professionals not elsewhere classified, Health care assistants, Home-based personal care workers, Hospitality, retail and other services managers, Hotel and restaurant managers, Hotel managers, Hotel receptionists, Housewife, Human resource managers, Journalists, Judges, Kitchen helpers, Labourers in mining, construction, manufacturing and transport, Landscape architects, Lawyers, Legal professionals, Legal professionals not elsewhere classified, Legal secretaries, Legal, social and cultural professionals, Legal, social and religious associate professionals, Legal, social, cultural and related associate professionals, Legislators, Legislators and senior officials, Librarians and related information professionals, Librarians, archivists and curators , Library clerks, Lifting truck operators, Livestock and dairy producers, Livestock farm labourers, Mail carriers and sorting clerks, Manufacturing labourers, Medical records and health information technicians, Medical secretaries, Messengers, package deliverers and luggage porters, Midwifery associate professionals, Midwifery professionals, Musicians, singers and composers, Nursing and midwifery associate professionals, Nursing and midwifery professionals, Nursing associate professionals, Nursing professionals, Office supervisors, Other artistic and cultural associate professionals, Other arts teachers, Other cleaning workers, Other clerical support workers, Other language teachers, Other music teachers, Other personal services workers, Other sales workers, Painters and related workers, Painters, building structure cleaners and related trades workers, Pawnbrokers and money-lenders, Payroll clerks, Pelt dressers, tanners and fellmongers, Personal care workers, Personal care workers in health services, Personal care workers in health services not elsewhere classified, Personal services workers not elsewhere classified, Personal service workers, Personnel and careers professionals, Personnel clerks, Pet groomers and animal care workers, Pharmacists, Philosophers, historians and political scientists, Photographers, Plasterers, Police inspectors and detectives, Police officers, Policy administration professionals, Policy and planning managers, Poultry producers, Primary school and early childhood teachers, Primary school teachers, Prison guards, Product and garment designers, Protective services workers, Protective services workers not elsewhere classified, Psychologists, Public relations professionals, Real estate agents and property managers, Receptionists (general), Refuse sorters, Refuse workers, Refuse workers and other elementary workers, Regulatory government associate professionals, Regulatory government associate professionals not elsewhere classified, Religious associate professionals, Religious professionals, Restaurant managers, Retail and

wholesale trade managers, Sales and marketing managers, Sales and purchasing agents and brokers, Sales demonstrators, Sales, marketing and development managers, Sales, marketing and public relations professionals, Sales workers, Sales workers not elsewhere classified, Scribes and related workers, Secretaries (general), Security guards, Senior government officials, Service and sales workers, Services managers not elsewhere classified, Service station attendants, Shelf fillers, Shop keepers, Shop sales assistants, Shop salespersons , Shop supervisors, Social and religious professionals, Social welfare managers, Social work and counselling professionals, Social work associate professionals, Sociologists, anthropologists and related professionals, Special needs teachers, Sports and fitness workers, Sports coaches, instructors and officials, Sports, recreation and cultural centre managers, Stall and market salespersons, Street and market salespersons, Street and related sales and service workers, Street and related service workers, Street food salespersons, Street vendors (excluding food), Subsistence farmers, fishers, hunters and gatherers, Subsistence fishers, hunters, trappers and gatherers, Subsistence livestock farmers, Subsistence mixed crop and livestock farmers, Survey and market research interviewers, Sweepers and related labourers, Tellers, money collectors and related clerks, Trade brokers, Traditional chiefs and heads of village, Training and staff development professionals, Translators, interpreters and other linguists, Transport and storage labourers, Transport clerks, Transport conductors, Travel attendants and travel stewards, Travel attendants, conductors and guides, Travel consultants and clerks, Travel guides, Tree and shrub crop growers, Typists and word processing operators, Undertakers and embalmers, Underwater divers, Upholsterers and related workers, Valuers and loss assessors, Vehicle cleaners, Vehicle, window, laundry and other hand cleaning workers, Veterinarians, Veterinary technicians and assistants, Waiters, Waiters and bartenders, Water and firewood collectors, Window cleaners.

References

OECD (2014). PISA 2012: Technical Report. OECD Publishing, Paris.

Stevenson, H. W., Lee, S. Y., Chen, C., Stigler, J. W, Hsu, C. C., & Kitamura, S. (1990). Contexts of achievement: A study of American, Chinese and Japanese children. *Monographs of the Society for Research in Child Development, 55* (Serial No. 221).
